# Are All Code-Switches Processed Alike? Examining Semantic v. Language Unexpectancy

**DOI:** 10.3389/fpsyg.2020.02138

**Published:** 2020-09-03

**Authors:** Jorge R. Valdés Kroff, Patricia Román, Paola E. Dussias

**Affiliations:** ^1^Department of Spanish and Portuguese Studies, University of Florida, Gainesville, FL, United States; ^2^Department of Psychology, Universidad Loyola Andalucía, Seville, Spain; ^3^Department of Spanish, Italian, and Portuguese, The Pennsylvania State University, State College, PA, United States

**Keywords:** code-switching, semantic processing, event-related potentials, late positive complex, N400, bilingual (Spanish/English)

## Abstract

Prior studies using the event-related potential (ERP) technique show that integrating sentential code-switches during online processing leads to a broadly distributed late positivity component (LPC), while processing semantically unexpected continuations instead leads to the emergence of an N400 effect. While the N400 is generally assumed to index lexico-semantic processing, the LPC has two different interpretations. One account suggests that it reflects the processing of an improbable or unexpected event, while an alternative account proposes sentence-level reanalysis. To investigate the relative costs of semantic to language-based unexpectancies (i.e., code-switches), the current study tests 24 Spanish-English bilinguals in an ERP reading study. Semantically constrained Spanish frames either varied in their semantic expectancy (high vs. low expectancy) and/or their language continuation (same-language vs. code-switch) while participants’ electrophysiological responses were recorded. The Spanish-to-English switch direction provides a more naturalistic test for integration costs to code-switching as it better approximates the code-switching practices of the target population. Analyses across three time windows show a main effect for semantic expectancy in the N400 time window and a main effect for code-switching in the LPC time window. Additional analyses based on the self-reported code-switching experience of the participants suggest an early positivity linked to less experience with code-switching. The results highlight that not all code-switches lead to similar integration costs and that prior experience with code-switching is an important additional factor that modulates online processing.

## Introduction

Over the last decade, interest in the psycholinguistic processes underlying the integration of code-switched speech, defined as the fluid alternation of both languages within the same conversation or in text (Poplack, 1980), has grown rapidly. There are now several reviews dedicated to this topic (Van Hell et al., 2015, 2018; Beatty-Martínez et al., 2018; Valdés Kroff et al., 2018) building off of prior and more established work by sociolinguists and structural linguists (see Bullock and Toribio, 2009; Gardner-Chloros, 2009 for comprehensive reviews). Yet, the processing of code-switched speech remains understudied in comparison. Broadly speaking, much of the early work on code-switching indicates that, just like in task switching (Monsell, 2003) and cued-language switching (Meuter and Allport, 1999), integrating code-switches in real-time processing leads to greater switch costs relative to unilingual processing (Altarriba et al., 1996; Litcofsky and Van Hell, 2017). Nevertheless, recent available literature has revealed that switch costs may be attenuated under certain social or linguistic contexts (Fricke et al., 2016; Guzzardo Tamargo et al., 2016; Beatty-Martínez and Dussias, 2017; Valdés Kroff et al., 2018). One plausible account for the discrepancy between the ubiquity of code-switching in bilingual speech and the cognitive costs of its integration in comprehension is its unexpectancy in lab-based studies. As a means to providing a more complete picture, the study we report here builds off of prior work (Altarriba et al., 1996; Moreno et al., 2002) to directly compare different forms of unexpectancy: semantic and language-based (i.e., code-switches) unexpectancies.

Critically, linguists have demonstrated that code-switching is not a random or chaotic process, and that instead it is systematic and constrained (Poplack, 1980; Myers-Scotton, 1993). Linguists draw on a distinction between two types of code-switches by taking the complementizer phrase (CP) as the major delineation between both types: switches that occur between the CP are known as inter-sentential (or clausal) code-switches (e.g., *Fui al supermercado, and I bought some milk* “I went to the store and I bought some milk”), whereas those that occur within the CP are typically classified as intra-sentential (or clausal) code-switches (e.g., *El niño está reading the book* “The boy is reading the book”). Although the search for grammatical constraints that can universally account for code-switching patterns remains elusive, this distinction is important because individual- and community-level factors affect the type of code-switching structure produced, as well as the frequency with which individuals will engage in code-switching.

Due to the heterogeneity of bilingual acquisition, proficiency in the component languages is one such individual-level factor. Higher proficiency bilinguals are more likely to engage in intra-sentential code-switches, whereas lower proficiency bilinguals are more restricted to inter-sentential and single-word code-switches (Miccio et al., 2009). Similarly, not all bilingual communities frequently code-switch. In a remarkable demonstration of community-determined code-switching patterns, Poplack (1988) analyzed bilingual speech from Spanish-English bilinguals in New York City and French-English bilinguals from the Ottawa-Hull region of Canada. Despite the similarity of language pairs involved, Poplack found that Spanish-English bilinguals produced more frequent and more varied code-switches as compared to the French-English bilinguals, who restricted their code-switching patterns to single-word switches and “tagged” switching (i.e., fixed phrases).

The current psycholinguistic studies of code-switching highlight three broad themes of study: (1) Its relationship to other switching phenomena such as cued-language switching (e.g., Meuter and Allport, 1999; Gollan and Ferreira, 2009) and non-linguistic switching tasks (e.g., Monsell, 2003); (2) whether the integration of code-switching in production and comprehension leads to processing costs (e.g., Ruigendijk et al., 2016; Beatty-Martínez and Dussias, 2017; Litcofsky and Van Hell, 2017; Fernandez et al., 2019); and (3) the cognitive and grammatical processes that help bilinguals rapidly integrate code-switched speech in production and comprehension (e.g., Kootstra et al., 2012; Fricke et al., 2016; Guzzardo Tamargo et al., 2016; Valdés Kroff et al., 2017; Gullifer and Titone, 2019; Adler et al., 2020). These three themes are inter-related in that the natural parallel between general switching behavior and the robust switch costs reported from the cued-language switching paradigm leads to the logical prediction that code-switching should similarly evince costly integration. In the discussion that follows, we focus on point (2) given its relevance to the goal of the present study.

Altarriba et al. (1996) is one of the first behavioral studies to investigate code-switching costs to integration (although the study was not framed as a code-switching study *per se*). Using naming times in a rapid serial visual presentation paradigm and fixation durations from eye-tracking while reading, Altarriba et al. (1996) examined the processing of same-language English target words or code-switched Spanish target words that varied in lexical frequency (high, low) and the semantic restrictiveness of the preceding sentential context (high constraint, low constraint). Critically, only code-switched words (i.e., Spanish target words in an otherwise English sentence) resulted in a frequency × constraint interaction, such that higher frequency words required increased processing time when they were embedded in high constraint sentences than in low constraint sentences. This asymmetric cost suggests that bilingual speakers experience more difficulty integrating code-switches when the sentential context leads the parser to anticipate highly expected information. Conversely, the slower processing of lower frequency conditions leads to more time to resolve conflict, thus attenuating potentially upcoming conflict costs experienced when encountering a code-switch.

At the neurocognitive level, Moreno et al. (2002) focused on high constraint sentential contexts that continued in a semantically expected same-language target word, a semantically unexpected but plausible same-language target word, or a translation into Spanish of the semantically expected continuation (effectively, a single-word code-switch from English into Spanish). Using the event-related potentials (ERPs) technique, Moreno et al. (2002) found that relative to the same-language expected completion, same-language unexpected continuations led to the emergence of the N400, an ERP component typically elicited over centro-parietal areas that indexes difficulty in lexico-semantic integration (Kutas and Hillyard, 1980; Gunter et al., 2000). In contrast, expected continuation code-switched targets elicited a late positivity component (LPC) over frontal-posterior areas. Broadly, Moreno et al. (2002, p. 202) interpreted this finding as indicative that code-switches do not reflect processing difficulties in semantic integration. Instead, they suggest that processing of code-switches, at least in the context of their study, reflected the processing of unexpected or improbable events. Additionally, they raised a relevant caveat for the current study, indicating that the code-switches were presented from English into Spanish although “bilingual speakers in the local community are more likely to code-switch from Spanish into English,” which may have induced a greater level of improbability.

Building from this seminal work, Van Hell and colleagues have continued to explore the individual-level factors that may contribute to the emergence of the N400 and the LPC as they relate to the processing of code-switches. Litcofsky and Van Hell (2017) examined how language dominance and language switch direction affect the processing of code-switches in Spanish-English bilinguals. Interestingly, they found that code-switches into a bilingual’s less dominant language led to an increased LPC, which they interpreted as reflective of sentence-level reanalysis. Code-switches into the more dominant language led to a weaker anterior negativity. The emergence of the LPC when code-switching into the weaker language was additionally found in the auditory domain (Fernandez et al., 2019) and in earlier studies (Ng et al., 2014; Ruigendijk et al., 2016; Beatty-Martínez and Dussias, 2017). More recently, Beatty-Martínez and Dussias (2017) reported an early frontal positivity (P2 or P3a) associated with prior experience with code-switching. Specifically, bilinguals who came from environments with little code-switching experience (Spain) showed this early frontal positivity whereas bilinguals from code-switching environments (U.S.) did not. Beatty-Martínez and Dussias interpret the early positivity as indicating an attentional shift from a more competitive to a more cooperative state of bilingualism (see Green and Wei, 2014; Green, 2018 for a corresponding theoretical model).

Following these important lines of research, the study reported here extends the paradigm first reported in Moreno et al. (2002) by directly comparing two forms of unexpectancy: semantic unexpectancy and code-switching (as a form of language-based unexpectancy). We extend the work of Moreno et al. (2002) by including a new condition missing in this early work; namely, a translation into Spanish of the semantically unexpected but plausible continuation. This addition will allow us to investigate whether all code-switches are processed similarly or if an increasingly unexpected code-switch results in greater processing difficulty. Furthermore, our code-switches will all be from Spanish to English to reflect the code-switching practices of U.S. Spanish-English bilinguals being tested here (Valdés Kroff et al., 2018). While we anticipate replicating the N400 effect for same-language unexpected continuations (e.g., Kutas and Hillyard, 1980), the code-switched conditions may result in different outcomes:

- If the LPC is linked to the processing of code-switches more generally, we should replicate Moreno et al. (2002) and find an LPC for the semantically-expected code-switched target, as well as for our new semantically-unexpected code-switched target.
- If the LPC, however, is linked to improbability, we should not find the LPC for the semantically-expected code-switched targets because the direction of the code-switch in our materials respects linguistic ecology (i.e., switches are from Spanish into English).
- Additionally, if the added semantic unexpectancy adds difficulty to semantic integration for the bilingual participants, we should find an N400 associated with the integration of the semantically-unexpected code-switched target.
- Finally, we will use self-reports on code-switching experience to investigate whether we find modulation of an early frontal positivity in our bilingual sample.

## Experiment

### Materials and Methods

#### Participants

Twenty-four Spanish-English highly proficient bilinguals (17 female; mean age = 23.83; *SD* = 4.34) participated for monetary compensation. Participants were students at a large US institution; all were right-handed and had normal or corrected vision. Responses on the LEAP-Q (Marian et al., 2007) revealed that participants were proficient in Spanish and English (Spanish, *M* = 9.17, *SD* = 1.53; English, *M* = 8.67, *SD* = 1.17, on a scale from 1, non-proficient, to 10, very proficient), and had begun learning both languages early in their lives (Spanish, mean age = 1, *SD* = 3.11; English, *M* = 6.13, *SD* = 0.88). Verbal fluency in both English and Spanish, and portions of the *Diploma de Español como Lengua Extranjera* (DELE) and the *Michigan English Language Institute College English Test* (MELICET) served as additional objective measures in assessing participants’ level of vocabulary and grammatical proficiency (Spanish verbal fluency, *M* = 48, *SD* = 13.35; English verbal fluency, *M* = 59, *SD* = 12.99; DELE score, *M* = 36 (out of 50), *SD* = 9.08; MELICET score, *M* = 36 (out of 50), *SD* = 7.64). Finally, participants completed a code-switching questionnaire (Dussias, 1997) and reported code-switching frequently within the same conversation (*M* = 2.33; *SD* = 0.48, on a scale from 1, never, to 3, often).

#### Stimuli

One hundred and sixty sentences constituted the materials in the reading task. All sentence contexts were semantically constrained and represented four conditions: (1) a sentence with a semantically expected same-language target word (same-language, expected continuation); (2) a sentence with a semantically unexpected but plausible same-language target word (same-language, unexpected continuation); (3) a sentence with the English translation of the semantically expected target word (code-switched, expected continuation); and (4) a sentence with the English translation of the semantically unexpected target word (code-switched, unexpected continuation). Sample stimuli are provided in Table 1.

**Table 1:** Example stimuli.

	Same-language (Spanish) continuation	Code-switched continuation
Highly expected target	Los jóvenes se reunieron para ver el partido y apoyar al equipo. “The guys got together to watch the game and to support the team.”	Los jóvenes se reunieron para ver el partido y apoyar al team. “The guys got together to watch the game and to support the team.”
Low expected target	Los jóvenes se reunieron para ver el partido y apoyar al entrenador. “The guys got together to watch the game and to support the coach.”	Los jóvenes se reunieron para ver el partido y apoyar al coach. “The guys got together to watch the game and to support the coach.”

Frequency (log frequency from NIM database, Guasch et al., 2013) and length across the four types of target words (all nouns) were not significantly different as confirmed by ANOVAs (Frequency: *F*_expectancy_ = 1.22 *p* = 0.28; *F*_language_ = 0.03, *p* = 0.86; *F*_expectancyxLanguage_ = 3.31, *p* = 0.08; Length: *F*_expectancy_ = 0.76, *p* = 0.39; *F*_language_ = 0.161 *p* = 0.21; *F*_expectancyxLanguage_ = 1.47, *p* = 0.23). Mean values and standard deviations are reported in Table 2.

**Table 2:** Frequency and length values for critical nouns.

		Frequency		Length	
		*M*	*SD*	*M*	*SD*
High cloze	Spanish	1.58	0.60	6.70	2.10
	English	1.44	0.69	5.80	2.31
Low cloze	Spanish	1.31	0.57	6.45	1.92
	English	1.49	0.59	6.48	2.42

Sentence completion norms were collected using a cloze procedure in order to verify the semantic constraint of the experimental materials. To this end, the 160 experimental sentences were truncated immediately before the target word. Twenty-six Spanish-English bilinguals who did not take part in the main study (15 females; mean age = 36.64, *SD* = 11) were asked to complete the sentences in Spanish using a single-word that they felt best completed the sentences. Data were collected using Amazon’s Mechanical Turk^1^. The 26 participants in the norming study completed the LEAP-Q (Marian et al., 2007) and a code-switching questionnaire (Dussias, 1997) to verify that their linguistic characteristics and language experience were similar to Spanish-English speakers who participated in the experiment proper. The average cloze frequency for high constraint completions was at least .67 (Block and Baldwin, 2010). Norming materials and frequency completions are included in Supplemental Materials available at the Open Science Framework (OSF) repository associated with this project^2^.

Each of the four versions of a sentence was randomly assigned to one of four experimental lists. The lists were comprised of 40 sentences per condition for a total of 160 experimental trials. Participants read only one of the lists in the procedure. Twenty “catch trial” sentences (10 unilingual Spanish, 10 with a single-word insertion from English) describing horse-related content were included to each list to ensure that participants were paying attention to the task (see “Procedure”). Samples sentences are provided below:

1. Catch trial-unilingual: *Me contaron que se puede encontrar una isla en la costa con caballos salvajes*. (“I was told that one can find an island on the coast with wild horses”).
2. Catch trial-code-switched: *Una curiosidad de los caballos es que solamente pueden ver tres colores: verde, amarillo y gray*. (“An interesting fact about horses is that they can only see three colors: green, yellow, and gray”).

#### Procedure

Participants were seated in the recording chamber at a distance of 3.5 ft away from a 19-in LCD monitor (60 Hz refresh rate) enclosed in a Faraday cage to minimize electrical noise (Luck, 2015) and connected to a Dell Optiplex 755 computer (Intel Core 2 Processor; OS Windows XP Professional). The sentences were presented with E-Prime 2.0 Professional software (Psychology Software Tools Inc.; Schneider et al., 2002) one word at a time using a rapid visual serial presentation paradigm. Each word was displayed at the center of the screen using a black 24-point Courier New font on an opaque silver background (RGB 192,192,192). A trial started with a fixation cross (1,000 ms) preceding each sentence. Every word in the sentence stayed on the screen for 450 ms, followed by a blank screen (250 ms) until the next word appeared. After the last word of the sentence (the critical word) was displayed, a blank screen was presented for 500 ms. Participants were instructed to press the “y” key whenever they read a sentence about horses (catch trials). Incidence of catch trials was not predictable; thus, successful performance on catch trials indicated semantic processing and attention to task during the experimental session.

##### EEG Recording and Preprocessing

The electrophysiological activity of the brain (EEG) was recorded during the experimental task from 32 electrodes mounted on an elastic cap. The location of the electrodes was based on the 10/20 International System (Jasper, 1958). Four more electrodes were placed to monitor eye movements – two at the outer canthus of each eye, and one above and one below the left eye. An electrode on the right mastoid served as an online reference, and another electrode was placed on the left mastoid for offline re-referenciation to the average of the two mastoids. The electrophysiological signal was amplified with a 0.05 high pass filter and a 100 Hz low pass filter and digitized at a sample of 500 Hz utilizing NeuroScan equipment (Synamps; Compumedics, El Paso, TX). Electrode impedances were kept below 5 kΩ.

EEG data were processed with Edit 4.3 software (Compumedics, El Paso, TX). The processing of the data consisted of the following steps: (1) visual inspection of the continuous signal and rejection of sections with artifacts, (2) eve-movement corrections, employing the spatial filter transform implemented in Edit 4.3 (Berg and Scherg, 1994), and (3) application of a 0.1–30 Hz band-pass filter offline. We cut epochs locked to the target words from −200 to 750 ms. The 200 ms before the target word were used to correct the epoch baseline. Epoch amplitudes that exceeded 50 μV above or below the baseline were not included in the analyses. As a result, an average of 52.95% of trials was removed after artifact rejection (±50 μV), and two participants were excluded due to a noisy signal in which they failed to register any data for at least one condition. While the percentage of rejected trials is high, we included a high number of sentences per condition (40 sentences per condition) to accommodate such a possibility while minimizing fatigue due to the time needed to complete the task (see Boudewyn et al., 2018 for further discussion regarding number of trials required to get a significant ERP effect). We suspect that the high rate may in part have been due to critical words occurring at the end of the sentence, leading to higher blink rates.

**Figure 1:**
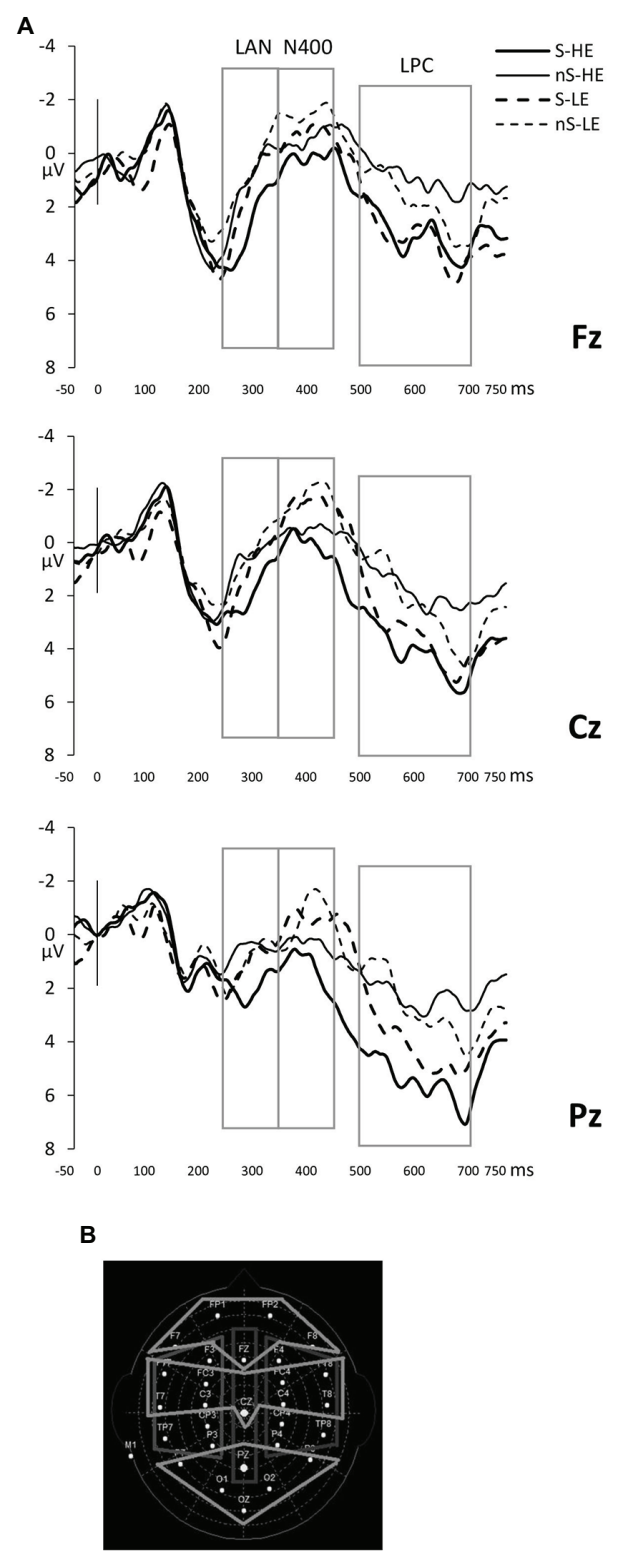
**(A)** Event-related potentials (ERPs) locked to the noun in electrodes representative of each area of interest in the midline: Fz for the frontal, Cz for the central, and Pz for the posterior regions. Targets that are not switched (nS) are depicted with a dashed line when they are semantically low expected (LE) and with a solid line when they are semantically highly expected (HE); for nouns that are code-switched (S), LE nouns are depicted with a bold dashed line and HE nouns with a bold solid line. Boxes indicate time windows included in the analyses. **(B)** Electrodes grouped in two topographical factor regions of Laterality (left, midline, and right electrodes) and Anterior/Posterior (anterior, central, and posterior electrodes) included in the analyses.

After visual inspection of the epochs and following prior studies on the processing of code-switches (Moreno et al., 2002; Proverbio et al., 2004; Ng et al., 2014), three time windows within the epochs were selected for further analyses. The time windows targeted the left anterior negativity (LAN, between 250 and 350 ms after the target display), the N400 (350–450 ms), and the late positivity complex (LPC, 500–700 ms). Because we were especially interested in the interaction between semantic unexpectancy and code-switches, we ran repeated-measures ANOVAs on the mean amplitudes for every time window, including the factors language continuation (Language: Spanish vs. code-switching), word expectancy (Expectancy: high vs. low), and two topographical factors to explore distribution of the neurophysiological data (see Figure 1B): Anterior/Posterior factor (anterior electrodes vs. central electrodes vs. posterior electrodes) and Laterality (left vs. midline vs. right). Results show corrected probabilities (Greenhouse/Geisser correction, Greenhouse and Geisser, 1959).

## Results

### Catch Trials

Participants responded correctly to 95.51% of catch trials (*SD* = 0.07) demonstrating that they were processing the content of the sentences semantically.

### Event-Related Potentials

#### Left Anterior Negativity (250–350 ms)

Neither the main effects of Language or Expectancy nor the interactions reached significance in this early time window (all *p*s > .05). The expectancy effect was marginally significant [*F*(1,21) = 3.34, MSe = 31.22, *p* = 0.082, *η_p_*^2^ = 0.14; observed power = 0.42] and manifested itself as a negativity to low-expected words. A marginally significant language effect [*F*(1,21) = 3.78, MSe = 3.07, *p* = 0.078, *η_p_*^2^ = 0.14; observed power = 0.42] showed a neurophysiological fluctuation associated with a code-switch that was positive rather than negative (Figure 1; grand averages across the scalp are found in Supplemental Materials at the OSF repository^3^), potentially suggesting an early frontal positivity (Beatty-Martínez and Dussias, 2017). We follow-up on this potential positivity in section *Experience Modulated Effects in the P300 Window (250-350 ms)*.

#### N400 (350–450 ms)

**Figure 2:**
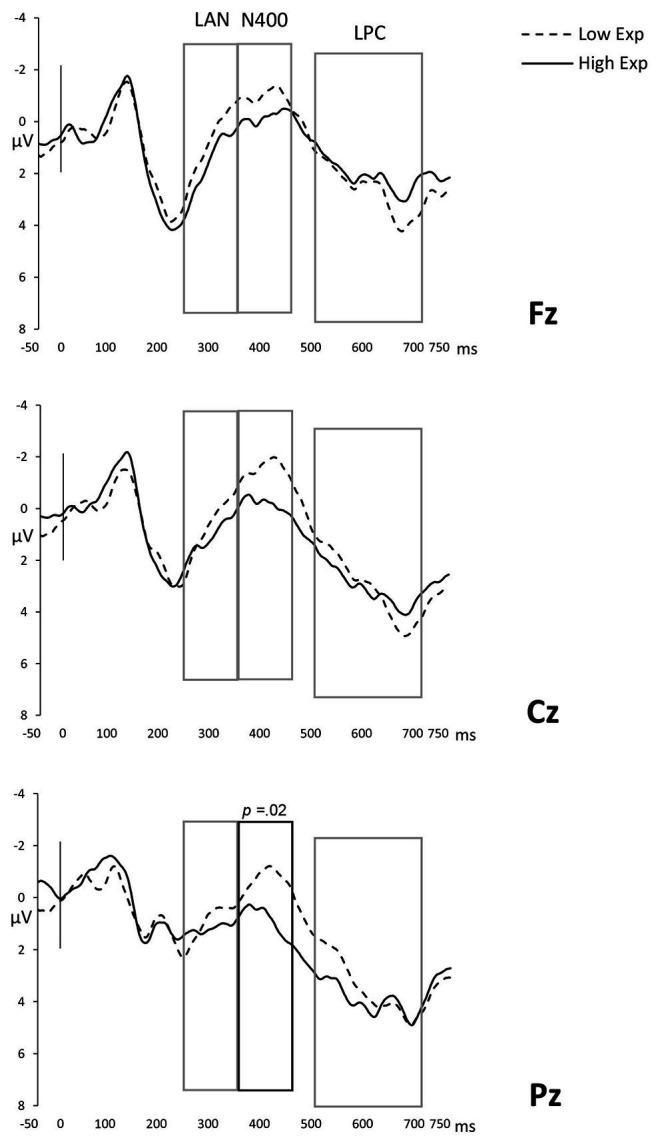
The Expectancy effect in Fz, Cz, and Pz electrodes. Boxes indicate time windows included in the analysis and dark outlines the regions where the effect is statistically significant.

The ANOVA on the mean amplitudes corresponding to this time window showed a main effect of Expectancy [*F*(1,21) = 4.33; MSe = 42.92; *p* = 0.04; *η_p_*^2^ = 0.08; observed power = 0.51], of the Anterior/Posterior factor [*F*(1,21) = 5.32; MSe = 11.26; *p* = 0.02; *η_p_*^2^ = 0.20; observed power = 0.65] and Laterality [*F*(1,21) = 4.01; MSe = 6.06; *p* = 0.03; *η_p_*^2^ = 0.16; observed power = 0.62], as well as an Expectancy × Anterior/Posterior factor interaction [*F*(1,42) = 4.42; MSe = 11.13; *p* = 0.04; *η_p_*^2^ = 0.17; observed power = 0.56]. No other comparisons were significantly different (*p*s > .10). A closer inspection of the Expectancy effect revealed that low-expected nouns presented greater negativity compared to highly expected ones (low-expectancy, *M* = −0.90, *SD* = 2.03; high-expectancy, *M* = 0.07, *SD* = 2.07), reflecting an N400 fluctuation in response to our semantic expectancy manipulation regardless of the use of the same-language or a code-switch in the critical word (see Figure 2). These results suggest that the N400 is, under these circumstances, a component associated with lexico-semantic integration and is not directly related to code-switching. Similarly, code-switches that involve unexpected continuations are harder to integrate semantically, further distinguishing between code-switches based on semantic content. To uncover the topographical distribution of the N400, we explored the Expectancy × Anterior/Posterior interaction. Planned comparisons showed that the N400 component was located in the posterior electrodes [*F*(1,21) = 6.86; MSe = 31.94; *p* = 0.02] but not in central or anterior regions (*p*s > .05). This pattern is consistently found in the literature on the N400 with linguistic and non-linguistic materials (see Kutas and Federmeier, 2011, for a review).

#### Late Positivity Component (500–700 ms)

**Figure 3:**
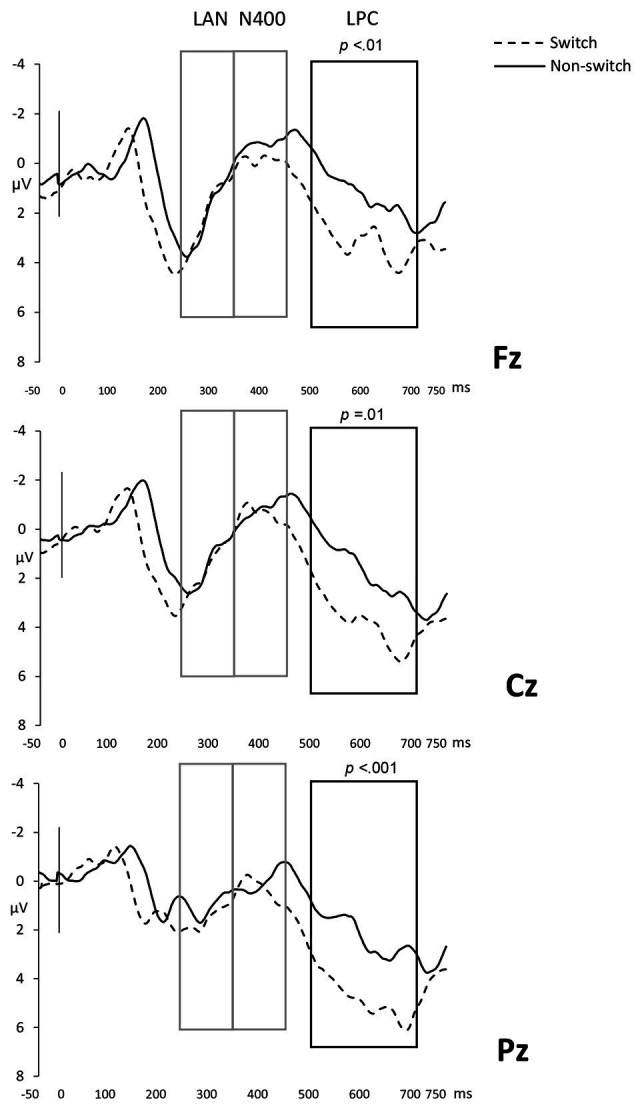
Switched vs. non-switched conditions in Fz, Cz, and Pz electrodes. Boxes indicate time windows included in the analysis and dark outlines the regions where the effect is statistically significant.

A late positivity arose in response to language continuation but not Expectancy as revealed by a main effect of Language [*F*(1,21) = 21.65; MSe = 21.39; *p* < .001; *η_p_*^2^ = 0.51; observed power = 0.99]. Code-switched words elicited an increased positivity compared to the same-language conditions (code-switched, *M* = 3.21, *SD* = 1.66; same-language, *M* = 1.68, *SD* = 1.53). Because the main effect of the two topographical factors and the first order Language × Anterior/Posterior interaction was significant [Anterior/Posterior, *F*(1,21) = 11.09; MSe = 13.36; *p* < .01; *η_p_*^2^ = 0.35; observed power = 0.92; Laterality, *F*(1,21) = 5.21; MSe = 7.45; *p* = 0.02; *η_p_*^2^ = 0.20; observed power = 0.72; and interaction, *F*(1,42) = 4.03; MSe = 6.53; *p* = 0.045; *η_p_*^2^ = 0.16; observed power = 0.55], we carried out further planned comparisons to locate the LPC effect. Unlike the N400 component, the LPC was greater for code-switched words in all regions [anterior, *F*(1,21) = 9.49, *p* > .01; central, *F*(1,21) = 13.19, *p* = 0.01; and posterior, *F*(1,21) = 17.97, *p* < .001]. A more detailed comparison of the effect across regions showed significant differences in amplitude between anterior and posterior regions (*F* = 4.43; MSe = 3.91; *p* = 0.047), thus indicating code-switches may cause an extended LPC that is more accentuated in posterior electrodes (see Figure 3). Robust evidence for the LPC for code-switched trials indicate that, at least in the context of the experimental stimuli, LPC is broadly reflective of sentence reanalysis (Litcofsky and Van Hell, 2017) and not just event improbability (Moreno et al., 2002).

#### Planned Comparisons of Code-Switched, Unexpected Continuations

**Figure 4:**
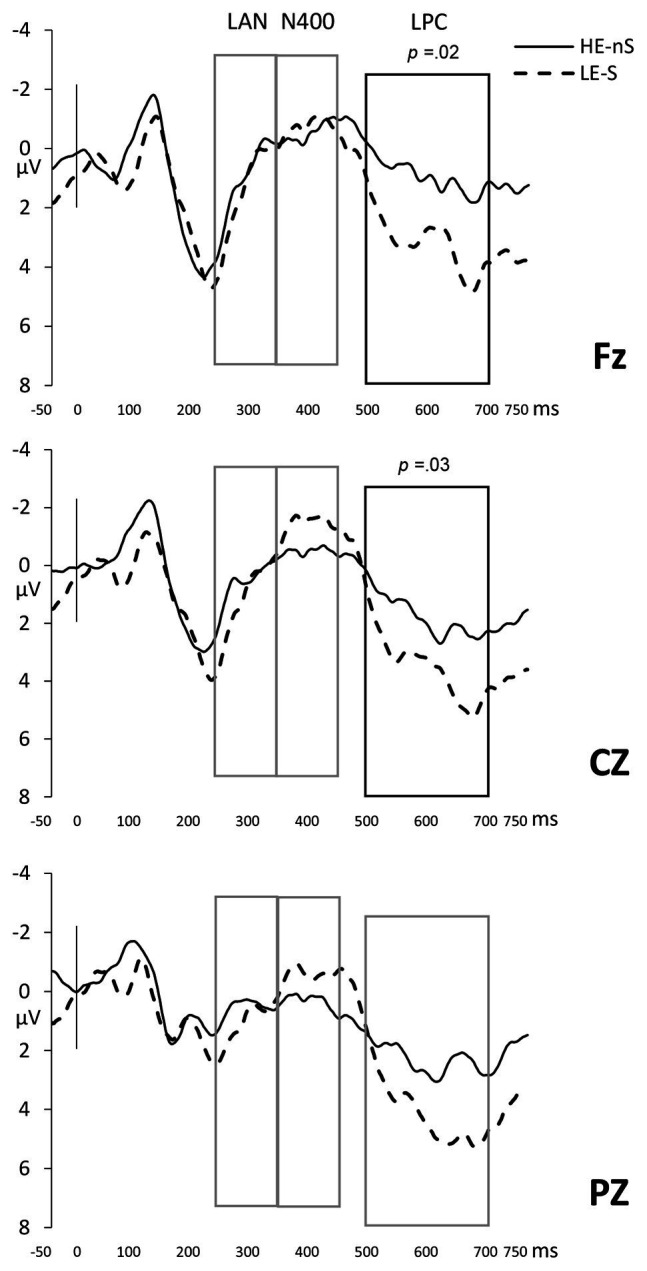
The baseline (non-switched, highly expected continuation) vs. the double unexpectancy (code-switched and semantically unexpected continuation) in Fz, Cz, and Pz electrodes. Boxes indicate time windows included in the analysis and dark outlines the regions where the effect is statistically significant.

Despite the lack of a significant Expectancy × Language interaction in the ANOVAs above, we directly compared the combination of semantic unexpectancy and code-switches to highly expected same-language continuations. We compared the mean amplitudes of the baseline condition (same-language, expected continuation) to those belonging to our new critical condition (code-switched, unexpected continuation) in the N400 and LPC time windows. Because the results show evidence for an N400 for the Expectancy manipulation and an LPC for the Language manipulation, we expected to observe a combination of an N400 and an LPC to the critical condition as a result of the combination of the two forms of unexpectancy relative to the baseline condition. However, the planned comparisons of the two conditions only unveiled significant brain response differences in the time window corresponding to the LPC, between 500 and 700 ms post noun (main effect *F* = 4.61, MSe = 56.31, *p* = 0.04; *F*s < 1 for early and N400 time windows). The code-switched, unexpected continuation condition evinced a late positivity compared to the same-language, expected continuation condition that presented a wide distribution in the scalp, but was significant only for anterior and central regions [anterior: *t*(21) = 2.66, *p* = 0.02; central: *t*(21) = −2.40, *p* = 0.03; see Figure 4]. A potential explanation for the lack of an N400 to the code-switched, unexpected continuation is the combination of the positive trend in the early time window and the LPC, both to code-switches, counteracting the negativity in the 350–450 ms range.

#### Experience Modulated Effects in the P300 Window (250–350 ms)

To test whether prior code-switching experience affected the emergence of an early positivity between 250 and 350 ms, we split the sample into two subgroups based on their self-reported code-switching experience. Although the number of participants is limited, the subgrouping led to a group who reported to code-switch “often” (*n* = 8) and another subgroup who only indicated to code-switch “sometimes” (*n* = 14). The ANOVA including code-switching frequency as a grouping factor did show a group effect [*F*(1,20) = 4.92; MSe = 93.12; *p* = 0.04; *η_p_*^2^ = 0.20; observed power = 0.56]. No other result was significant for the early time-window (all *p*s > .1). Separated ANOVAs per group showed no effect for the “often” subgroup (all *p*s > 0.1) but the “sometimes” group did show a marginally significant switch effect [F(1,13) = 4.22; MSe = 30.61; *p* = 0.065; *η_p_*^2^ = 0.24; observed power = 0.46], hinting at the emergence of an early positivity for code-switched conditions. While sample size is low, this trending early positivity follows recent suggestions in the literature on code-switching habits affecting the processing of code-switches (Beatty-Martínez and Dussias, 2017).

## Discussion

In this study, we sought to replicate and extend one of the first ERP studies on the online processing of code-switching by Moreno et al. (2002). In their original study, semantically constraining English sentential frames varied in their completions, which ended in a same-language expected continuation, a same-language unexpected but plausible continuation, or a code-switched Spanish continuation of the expected target. We included two changes to the original design. As pointed out in the original study, code-switching from English into Spanish is a less ecological switch direction for Spanish-English bilinguals residing in the U.S. (Moreno et al., 2002; Valdés Kroff et al., 2018); consequently, we included semantically constraining sentences that started in Spanish. Additionally, we included a new experimental condition that code-switched into English and included an unexpected but plausible continuation, which resulted in a 2 (Language) × 2 (Expectancy) factorial design. With this updated design, our goals were to investigate whether we would find the LPC in our code-switched conditions, originally interpreted as possibly reflecting the processing of an improbable or unexpected event, and whether the addition of a semantically unexpected component to a code-switch would in turn be reflected by the emergence of an N400, as found in the same-language unexpected condition. Finally, we investigated whether prior code-switching experience would modulate an early positivity component (Beatty-Martínez and Dussias, 2017).

Our findings broadly replicate the original Moreno et al. (2002) study. We found a main effect of Expectancy in the N400 time window, suggesting greater processing difficulty for lexico-semantic integration. The N400 is a robust effect found in both L1 and L2 processing and across a variety of tasks (e.g., Kutas and Hillyard, 1980; Kutas and Federmeier, 2011). Our novel contribution is to extend this semantic effect to code-switched contexts. Previously, Beatty-Martínez and Dussias (2017) also report an N400 effect to grammatical gender incongruent code-switches, only in Spanish-English bilinguals exposed to habitual code-switching. Here, we demonstrate that bilingual readers experience greater processing difficulty when sentence continuations do not match an expected sentence completion.

Moreover, we report a broadly distributed and extended LPC for the code-switched conditions. The LPC has now been documented in several studies on code-switching (e.g., Moreno et al., 2002, 2008; Ng et al., 2014; Ruigendijk et al., 2016; Beatty-Martínez and Dussias, 2017; Litcofsky and Van Hell, 2017; Fernandez et al., 2019; Kaan et al., 2020). While Moreno et al. initially interpreted this component as reflective of processing an improbable or unexpected event, Van Hell and colleagues have suggested that it instead points toward sentence-level reanalysis. By creating sentence materials that start in Spanish and code-switch into English, we tested whether increasing the probability of a code-switch (by making the switch direction ecologically more similar to the code-switching habits of Spanish-English bilinguals in the U.S.) would result in an elimination of the LPC, at least in conditions that fit the semantic expectation of the sentence frame. Nevertheless, the LPC was found for code-switch conditions. This finding is compatible with the interpretation of the LPC as reflecting sentence-level reanalysis. Alternatively, while we argue that the sentence materials are more ecologically similar to U.S. code-switching practices, the experimental context remains artificial in that stimuli are presented visually and without a supporting interactive exchange, while code-switching remains primarily a spoken language phenomenon rooted in dialogic exchange. Fernandez et al. (2019) used the ERP technique to test the processing of auditory code-switched sentences. Interestingly, for a subset of code-switches they do not find an LPC effect but instead an N400 effect; however, they frame their study in terms of language dominance and switch direction and not in habitual code-switching practices. Ruigendijk et al. (2016) similarly used auditory stimuli in a group of late Russian-German bilinguals. In code-switches from L2 German into L1 Russian, they also find the LPC but modulated by L2 proficiency such that increasing L2 proficiency leads to reduced LPC amplitudes.

Although our results revealed a strong N400 effect for unexpected continuations, the direct planned comparison between our baseline condition (same-language, expected continuation) and the code-switched, unexpected continuation, only evinced an LPC effect and not an N400 effect. The lack of an N400 effect may be due to statistical power, especially since the bilingual sample did show some variation in their own code-switching experience or may be due to the conflation of a possible N400 effect, as visually suggested in Figures 1A, 4, with the later and stronger positivity component. Future studies that include a greater number of habitual code-switchers may be able to tease these effects apart even further.

Following recent results suggesting that an early positivity component may be tied to prior experience with code-switching (Beatty-Martínez and Dussias, 2017), we explored whether a group-split analysis would similarly show a modulation of this early effect. While the effect was marginal, likely due to small numbers of participants in each subgroup, the trend was in the predicted direction such that the early positivity for code-switch conditions was suggestive in the group that reported “sometimes” code-switching while absent in the group that reported “often” engaging in code-switching practices. Beatty-Martínez and Dussias interpreted this early positivity as a switch cost resulting from a need to engage in an attentional shift from a “narrower” (i.e., more unilingual-like) attentional state to a “broader” attentional state. Although only suggestive, our replication of this recent finding highlights the need to incorporate code-switching experience into experimental studies on code-switching (Beatty-Martínez et al., 2018; Valdés Kroff et al., 2018).

## Conclusion

The current study used the ERP technique to directly compare two forms of unexpectancy: semantic unexpectancy with language-based unexpectancy. The results complement the now emerging picture from the nascent literature on the neurocognitive processes involved in the online processing of code-switching. Code-switches broadly elicit an LPC even when they match the code-switching patterns found in the targeted community of speakers. This late positivity suggests that the successful integration of code-switches requires sentence-level reanalysis. At the same time, additional factors, such as semantic expectancy and individual differences in exposure to code-switching, may affect the presence of additional neurocognitive processes. These findings suggest that not all sentential code-switches are processed with similar integration costs. Likewise, not all bilinguals experience similar integration costs. While these initial results require further replication, they point toward the increasing need to incorporate bilingual experience into experimental work on code-switching.

## Data Availability Statement

The datasets generated for this study are available at the Open Science Framework repository located at https://osf.io/py78j/.

## Ethics Statement

The studies involving human participants were reviewed and approved by The Pennsylvania State University Institutional Review Board. The participants provided their written informed consent to participate in this study.

## Author Contributions

JV, PR, and PD contributed to the conception and design of the study. JV, PR, and PD were involved in the implementation of the study. PR was involved in data collection and data preprocessing and statistical analysis. JV and PR wrote the first draft of the manuscript. All authors contributed to manuscript revision, and read and approved the submitted version.

### Conflict of Interest

The authors declare that the research was conducted in the absence of any commercial or financial relationships that could be construed as a potential conflict of interest.

## Footnotes

^1^www.Mturk.com

^2^https://osf.io/py78j/

^3^https://osf.io/py78j/
